# Addressing the First 90: A Highly Effective Partner Notification Approach Reaches Previously Undiagnosed Sexual Partners in Tanzania

**DOI:** 10.1007/s10461-017-1750-5

**Published:** 2017-03-15

**Authors:** Catherine Kahabuka, Marya Plotkin, Alice Christensen, Charlene Brown, Mustafa Njozi, Renatus Kisendi, Werner Maokola, Erick Mlanga, Ruth Lemwayi, Kelly Curran, Vincent Wong

**Affiliations:** 1 Jhpiego Tanzania, Dar es Salaam, Tanzania; 2 USAID Washington, Washington, DC USA; 3 National AIDS Control Programme, Ministry of Health, Community Development, Gender, Elderly and Children, Dar es Salaam, Tanzania; 4 USAID Tanzania, Dar es Salaam, Tanzania; 5 Jhpiego Baltimore, Baltimore, USA; 6 Johns Hopkins Bloomberg School of Public Health, Baltimore, USA; 7 1660 Thames Street, Baltimore, MD 21231 USA

**Keywords:** Partner notification, HIV testing services, Index clients, Undiagnosed HIV, Tanzania

## Abstract

To meet UNAIDS’ 90–90–90 treatment goals, effective approaches to HIV testing services (HTSs) are urgently needed. In 2015, a cross-sectional study was conducted to evaluate effectiveness and feasibility of partner notification for HTS in Tanzania. Men and women newly diagnosed with HIV were enrolled as index clients, listed sexual partners, and given options to notify and link their partners to HTS. Of 653 newly diagnosed individuals, 390 index clients were enrolled, listed 438 sexual partners, of whom 249 (56.8%) were successfully referred. Of 249 partners reaching the facilities, 96% tested for HIV, 148 (61.9%) tested HIV+ (all newly diagnosed), and 104 (70.3%) of partners testing positive were enrolled into HIV care and treatment. Results showed good acceptability, feasibility and effectiveness, as evidenced by high uptake of partner notification among newly diagnosed individuals, over half of listed partners successfully referred, and a very high positivity rate among referred sexual partners.

## Background

The proportion of people living with HIV (PLHIV) who know their status in sub-Saharan Africa (SSA) has risen from an estimated 10% in 2004 to 45% in 2015 [1, 2]. Effective approaches are urgently needed to find and diagnose the remaining 55% and link them to care and treatment, in support of UNAIDS’ 90–90–90 by 2020 goals [3]. In light of the clinical and prevention benefits of early initiation of treatment, the World Health Organization (WHO) now recommends early HIV case identification and early initiation of antiretroviral therapy (ART), regardless of CD4 count [4]. Partner notification—when partners of those recently diagnosed are notified of their exposure to a communicable disease—is an effective strategy to identify undiagnosed PLHIV and serodiscordant couples [5]. As an HIV testing services (HTSs) strategy, partner notification may contribute to prevention of onward HIV transmission, reduce HIV-related morbidity and mortality, and support epidemic control, particularly when combined with a “test and start” approach to ART in which all persons living with HIV are eligible to start treatment immediately [6].

With roots in sexually transmitted infection (STI) control and contact tracing, HIV partner notification is a process in which a person newly diagnosed with HIV, referred to as the “index client,” either contacts or has a health care provider contact his or her sexual partners to inform them of their HIV exposure and advise HIV testing. When health care providers conduct the notification, the provider notifies the partner of possible exposure without divulging the identity of the index client. If positive, partners are linked to HIV treatment services [7]. If negative, these partners may be at high risk of HIV infection and require additional prevention interventions if they remain in partnership with the index client. Partner notification is featured in the WHO 2015 consolidated guidelines on HTSs [8] and has been proven effective in identifying persons with undiagnosed HIV infection [9–11] but has been underutilized in SSA.

The following methods of partner notification described in the literature are relevant to this study:

- With passive referral, health workers encourage index clients to notify and refer their partners for HTS on their own (simple) [12], or with an invitation card or additional information (enhanced).
- Under contract referral, health workers encourage index clients to refer their partners for HIV testing, with the understanding that a health worker will contact partners who do not visit the site by an agreed-upon date.
- With provider referral, a trained health worker locates and notifies partners immediately and directly, while maintaining the anonymity of the index client [9, 13, 14].

Although HIV partner notification has long been established in the US and Europe [9, 15, 16], it has not been widely implemented in SSA [10, 11], and is not the standard of care in Tanzania. However, a growing evidence base supports its feasibility and effectiveness via facility- and community-based HTS programs [17], including prevention of mother-to-child transmission services [18] and STI clinics [10]. Recent HIV partner notification studies conducted in Malawi [10], Cameroon [11], and Mozambique [17] have consolidated the evidence supporting the feasibility [19] and acceptability of the passive, contract, and provider referral approaches to partner notification. A cluster-randomized trial in Kenya has also provided strong evidence for the success of provider-assisted partner notification [20].

The current study provides a unique contribution to the existing evidence on partner notification by examining the approach in the “real world” setting of routine, facility-based HTS in Tanzania. Our index clients presented at the health facility for voluntary counseling and testing (VCT) or were tested through provider-initiated testing and counseling (PITC). We gave index clients a choice of referral method, allowing us to document client preference with regard to partner notification, and test key outcomes with an eye to feasibility in the Tanzanian public health system. In this cross-sectional study, we assessed acceptability and measured effectiveness of partner notification in (a) locating and reaching high-risk sexual partners of index clients, (b) reaching a high proportion of undiagnosed HIV+ persons, (c) achieving successful linkage to treatment for HIV+ partners not currently in care, and (d) identifying serodiscordant couples.

## Methods

### Study Design and Setting

A cross-sectional study was conducted in three hospitals in Njombe region, Tanzania between June and September 2015. Njombe is Tanzania’s highest prevalence region where 14.8% of adults are infected with HIV [21]. Study facilities included peri-urban Kibena Regional Hospital, urban Makambako Town Hospital, and the rural, Faith-Based Ilembula Designated District Hospital. Each facility had a dedicated, onsite VCT center, and offered PITC to inpatients and outpatients. These three facilities were selected because of their high testing volume, in consultation with regional authorities.

### Study Population and Eligibility Criteria

Men and women newly diagnosed with HIV through VCT or PITC at the three study sites were screened for study eligibility. Eligibility criteria for index clients were: newly diagnosed with HIV, 18 years or older, not pregnant, had current sexual partner or had partner in the past 24 months. Pregnant women were excluded from the study since a form of partner services already exists within antenatal care services in Tanzania—pregnant women are requested to bring their sexual partner in for HIV testing. Referred sexual partners were enrolled in this study if they met the eligibility criteria for the study: 18 years or older, were listed as having been a sexual partner within the last 24 months, and had locator information, and consented to participate.

### Sample Size

We based our sample size calculation on an assumption that index clients would list an average of one sexual partner, and that 51% of partners would come to the facility following notification, as seen in the Malawi study [10]. Based on these assumptions, a sample size of 384 index clients was needed to detect a similar rate of attendance among sexual partners with 85% power (α = 0.05, two-sided test). The design effect (DEFF) was set at 1.0 because we expected minimal variation between facilities. The sample size formula for a single cross-sectional survey was:

$${\text{n}} = \frac{{1.96^{2} {\text{p}}(1 - {\text{p}})({\text{DEFF}})}}{{{\text{d}}^{2} }} = \frac{{1.96^{2} \times 0.51(1 - 0.51)(1.0)}}{{(0.05)^{2} }} = 384.$$

### Study Procedures

Individuals newly diagnosed with HIV through PITC or VCT were referred to onsite researchers, who were also HIV counselors, and screened for study eligibility. Written informed consent was obtained from interested and eligible participants, referred to as “index clients.” Enrolled index clients first answered a brief questionnaire that collected demographic information, general sexual history, history of intimate partner violence (IPV), and then were asked to list current or past (within 24 months) sexual partners. Clients with a history of IPV were noted, so that study staff could provide appropriate counseling. Written consent, separate from participation in the study, was obtained before the index client listed partners. Index clients were asked to list as many partners as they could, with locator information, duration, status (past or current) and type of relationship for each partner.

The study team member then informed the index client about the three types of partner notification (passive, contract, and provider) and the index client selected the preferred approach to notify each of the listed sexual partners. Partner notification by study staff was only initiated after obtaining consent from the index client. For passive referral, the study staff and the client agreed on a timeline when the index client would bring in or refer listed partners. Index clients received a pre-printed study referral card to give to partners, if they chose. If index clients did not bring in partners by the agreed date, study staff contacted the index client by phone to encourage him or her to complete the referral. For contract referral, the study staff initiated partner notification if after 2 weeks the index client had failed to bring in the sexual partner. For provider referral, the study staff contacted partners directly by phone within 24 h, and read pre-scripted information from the referral card, requesting partners to come for HTS. No information on the identity of the index client was provided to the partner. Study staff contacted partners three to five times before they were declared lost to follow-up (unless the partner declined the referral). Index clients were linked to partners using an ID code. Partners who came for services without the index client had been contacted in advance by the study team member and told where to come in the health facility, so the study team member was able to link the partner to the index client.

During partner listing, the study staff assisted index clients to assess the risk of IPV specific to each listed sexual partner, using a standardized set of questions. Any sexual partners the index client indicated might react with violence were excluded from the notification process.

Partners coming for HTS were informed of the study, consented, linked to the index client’s ID, and recorded as successful referrals. Unless already enrolled in an HIV Care and Treatment Centre (CTC), as verified by self-report or a CTC card, all sexual partners were offered HTS following the Ministry of Health, Community Development, Gender, Elderly and Children HIV testing protocols. Partners testing positive for HIV were referred to their chosen CTC using a referral form with a detachable portion which the client could return to the site of the original referral.

Three methods were used to verify study participants’ enrollment at the CTC; (1) returned note signed by CTC staff as confirmation of enrollment, (2) study staff checking CTC registers at the study facilities and at nearby facilities for the names and addresses of those who did not return the referral note, and (3) study staff contacting participants by phone and asking them whether they were already enrolled into CTC (self-report).

### Data Management and Analysis

Data were collected using both paper forms and electronic tablets. Paper-based data were entered into ODK data files that had field checks for data quality. Data collected using tablets were uploaded immediately to a server located in Dar es Salaam. Data were cleaned by running queries and reports using STATA version 14.0 and correcting discrepancies. Data were extracted and analyzed using SPSS version 23.

Descriptive statistics were performed to describe the background characteristics of index clients and successfully referred partners. Partners were considered successfully referred if they came to the respective facilities as a result of any notification method, whether or not they tested for HIV. Assessment of differences between sites was conducted and no major differences were seen on study outcome variables. Univariate and multivariate logistic regressions were run to identify predictors of participation in the study among newly diagnosed individuals, and success of referral among listed sexual partners. Backward elimination was used to establish the final logistic model. Covariates were included into the final model if they had a *p* value <0.25 and/or were known to affect the outcome of interest in previously published studies. Variables dropped out of the original model include: occupation, duration of relationship, and whether sexual partner is a current partner. Table 2 depicts variables that were retained in the final multivariate model and their effect on the study outcome.

### Ethical Considerations

The study was conducted with ethical oversight from the Institutional Review Boards (IRBs) of the Johns Hopkins University Bloomberg School of Public Health (IRB 00006116) and the Tanzania National Institute for Medical Research (NIMR/HQ/R.8a/vol.1x/1914) with support from the Njombe regional medical authorities.

## Results

### Study Overview

**Fig. 1:**
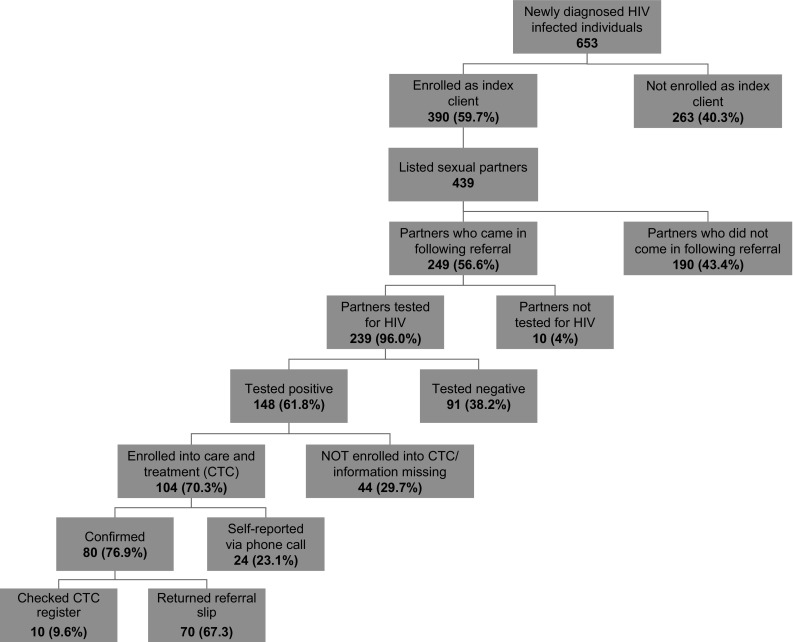
Overview of HTC partner notification study, Njombe, Tanzania, June–September 2015

Of the 653 individuals newly diagnosed with HIV who were approached about participation in the study, a total of 390 index clients were enrolled in the study (Fig. 1). A total of 263 (40.3%) newly diagnosed HIV+ individuals contacted for the study were not enrolled. The most common reason to not enroll in the study was the individual not having a sexual partner in the last 24 months (n = 167, 63.5%), followed by distraught or declined for other reason (n = 36, 13.6%), being under 18 years of age (n = 30, 11.4%), having insufficient contact information for partner (n = 11, 4.2%), being pregnant (n = 6, 2.3%), or other reasons (n = 13, 4.9%). The mean age of non-enrolled HIV+ individuals was similar to the mean age of enrolled index clients (32.2 vs. 33.2 years, respectively); however, the proportion of eligible males enrolled compared to females was higher (66.5% of males vs. 54.8% of females, p = 0.002), and the proportion of HIV+ individuals reporting they were single compared to those married was much lower (39.9% single vs. 82.3% married, p < 0.001; Table 1).

**Table 1:** Demographic characteristics of index clients and successfully referred sexual partners, Njombe, Tanzania, June–September 2015

Demographic factors	Index clients (n = 390)		Successfully referred sexual partners (n = 249)	
	Number	%	Number	%
Age groups				
18–24	62	15.9	41	16.5
25–34	174	44.6	96	38.6
35–44	102	26.2	70	28.1
45 and above	52	13.3	42	16.9
Sex				
Male	183	46.9	107	43.0
Female	207	53.1	142	57.0
Relationship status				
Single/never married	73	18.7	22	8.8
Married/living together	297	76.2	220	88.4
Divorced	14	3.6	5	2.0
Widowed	6	1.5	1	0.4
Missing information	0	0	1	0.4
Relationship status of listed sexual partners (classified by index client)				
Spouses (husband/wife)	–	–	206	82.7
Girlfriend/boyfriend	–	–	18	7.2
Casual sexual partner	–	–	20	8.0
Missing information			5	2.1
Level of education				
No formal education	62	15.9	55	22.2
Primary education	274	70.3	164	66.1
Secondary education and above	54	13.0	22	8.9
Main economic activity				
Housewife/house husband	6	1.5	3	1.2
Farmer	218	55.9	162	65.1
Small business/self-employed	126	32.3	65	26.1
Formally employed	40	10.3	18	7.2
Missing information	0	0	1	0.4
Total	390	100.0	249	100.0

The 390 index clients listed 439 sexual partners (average of 1.1 per index client). Initially, index clients chose passive referral for 402 (91.6%) partners, provider referral for 14 (3.2%) partners, and contract referral for 2 (0.5%) partners. Index clients refused partner notification services for 17 (3.9%) listed partners, and information on the selected referral approach was missing for four listed partners. In all but three cases, the approach the index client chose for the partner initially was successful in bringing in the partner for HTS: in two cases index clients chose provider referral but ended up bringing their partners themselves, and one client chose passive referral but then requested provider assistance.

Of the 439 listed sexual partners, 249 (56.7%) were successfully referred (came to the health facility); 242 (97.2%) through passive referral, 6 (2.4%) through provider, and 1 (0.4%) through contract referral. Of the successfully referred sexual partners, 239 (96.0%) were tested for HIV, of whom 148 (61.9%) tested HIV+. All of the partners testing HIV+ were newly diagnosed.

The 10 partners who came to the facility but were not tested had a previously confirmed HIV diagnosis, of which the index client was unaware. These 10 came to the facility with the index client and informed the client of their HIV status at the facility instead of testing. Of the HIV+ sexual partners, 104 (70.3%) were enrolled in HIV care and treatment by the end of the 3-month data collection period. Information on partner CTC enrollment was obtained through returned referral slip for 70 (67.3%), self-report over the phone for 24 (23.1%) and study staff checking area CTC registers for 10 (9.6%) of the sexual partners.

### Characteristics of Index Clients Who were Able to Successfully Refer at Least One Partner

Among index clients, nearly half (46.9%) were males and 76.2% were married (Table 1). Most index clients (70.3%) had completed primary education and 55.9% were farmers. Index clients successfully referred 206 (82.7%) sexual partners who were spouses, 18 (7.2%) who were boyfriend/girlfriend, and 20 (8.0%) who were casual partners. Among successfully referred sexual partners, 43.0% were males and 88.4% were married/cohabiting. The mean age was 33.2 years for index clients and 35.5 years for sexual partners (Table 1). Married index clients were 2.7 times more likely (CI 1.5–4.8) to successfully refer their sexual partners compared with unmarried index clients (Table 2). Women were less likely (OR 0.5, CI 0.3–0.7) to successfully refer at least one partner compared to men.

**Table 2:** Index clients who successfully referred at least one sexual partner, by background characteristics, Njombe, Tanzania, June–September 2015

Demographic factors	Index clients		OR (95% CI)	
	Total index clients (n = 390)	% successfully referred at least one partner	Univariate	Multivariate
Sex				
Male	183	71.0	Reference	
Female	207	51.7	0.4 (0.3–0.7)***	0.5 (0.3–0.7)**
Age (years)				
18–24	62	58.1	Reference	
25–34	174	58.0	1.0 (0.6–1.8)	0.6 (0.3–1.2)
35–44	102	62.7	1.2 (0.6–2.3)	0.6 (0.3–1.3)
45 and above	52	69.2	1.6 (0.7–3.5)	0.7 (0.3–1.7)
Marital status				
Single	73	41.1	Reference	
Married	297	66.7	2.9 (1.7–4.8)***	2.7 (1.5–4.8)**
Divorced	14	50.0	1.4 (0.5–4.5)	1.6 (0.5–5.2)
Widowed	6	33.3	0.7 (0.1–4.2)	0.8 (0.1–4.8)
Education levels				
No formal education	62	74.2	Reference	
Primary education	274	59.5	0.5 (0.3–0.9)*	0.5 (0.2–0.9)*
Secondary education or above	54	51.9	0.4 (0.2–0.8)*	0.4 (0.2–0.8)*

*OR* odds ratio, *CI* confidence interval

* p < 0.05, ** p < 0.01, *** p < 0.001

### HIV Testing, HIV Sero-discordance, and IPV

Among the tested sexual partners, women tested positive at a higher rate than men (67.2% women vs. 54.9% men, p = 0.036; Table 3). The highest HIV infection rate was seen among wives (69.3%), followed by casual partners (both male and female, 65.0%). Out of 233 couples who reported being in a current partnership, 88 were serodiscordant couples, i.e., the partner tested negative for HIV. No relationship was found between HIV positivity and relationship duration.

**Table 3:** HIV sero-status among tested sexual partners, Njombe, Tanzania, June–September 2015

Demographic factors	HIV sero-status of tested sexual partners n = 239		Total n (%)	p values
	HIV+ = 148 n (%)	HIV− = 91 n (%)		
Sex				
Male	56 (54.9)	46 (45.1)	102 (100.0)	
Female	92 (67.2)	45 (32.8)	137 (100.0)	0.036*
Relationship type (missing information = 5)				
Husband	42 (60.0)	28 (40.0)	70 (100.0)	
Wife	88 (69.3)	39 (30.7)	127 (100.0)	
Boyfriend/girlfriend	3 (17.6)	14 (82.4)	17 (100.0)	
Casual sexual partner	13 (65.0)	7 (35.0)	20 (100.0)	0.001*
Relationship duration (missing information = 5)				
Less than a year	22 (50.0)	22 (50.0)	44 (100.0)	
1–5 years	51 (63.7)	29 (36.3)	80 (100.0)	
6–10 years	29 (65.9)	15 (34.1)	44 (100.0)	
More than 10 years	44 (66.7)	22 (33.3)	66 (100.0)	0.297
Current sexual partner (missing information = 5)				
Yes	145 (62.2)	88 (37.8)	233 (100.0)	
No	1 (100.0)	0 (0.0)	1 (100.0)	0.624
Self-reported condom use in past 12 months among current sexual partners (missing information = 139)				
None	75 (60.0)	50 (40.0)	125 (100.0)	
Inconsistently	40 (71.4)	16 (28.6)	56 (100.0)	0.333
Consistently	8 (61.5)	5 (38.5)	13 (100.0)	

All 88 partners testing HIV− were described by the index clients as current sexual partners, meaning that the partner notification process found serodiscordant couples. Of the 71 HIV− current sexual partners whose information on condom use was recorded, 50 (70.4%) reported not using condoms at all, 16 (22.5%) reported using condoms inconsistently while only 5 (7.0%) said they used condoms consistently with the index clients during sex in the past 12 months (Table 3).

While six index clients indicated a general history of IPV in the index client interview form, no index clients indicated a perceived risk of IPV from a particular listed sexual partner (which would have disqualified that partner from notification; data not shown).

### Partner Referrals

Overall, 43.3% of partners did not come for HIV testing (Table 4). The most frequently cited reasons for not coming in for HIV testing included being geographically distant from the testing center (36.8%), either by residence or travel for livelihood reasons, followed by the contacted partner agreeing to come but not showing up (19.0%). Close to three-quarters (71.8%) of partners who came in for testing were escorted to HTS by the index client. The majority (61.7%) of partners came in for testing within 2 days of the index client enrollment (Table 4).

**Table 4:** Partner follow-up outcomes and process, Njombe, Tanzania, June–September 2015

Referral factors	n	%
Outcome of partner notification out of listed sexual partners		
Successfully referred	249	56.7
Not successfully referred	190	43.3
Total	439	100.0
Reasons for failure of referral		
Partner geographically distant (travel or residence)	70	36.8
Partner agreed to come but did not show up	36	19.0
Partner was not reached	29	15.3
Partner refused upon contact	18	9.5
Partner too busy to come in (farming/business/work)	9	4.7
Other reasons	28	14.7
Total	190	100.0
Partner escorted by index client (missing information = 1)		
Yes	178	71.8
No	60	24.2
Total	238	100.0
Days taken to successful referral (missing information = 1)		
Partner came to facility by day 2	153	61.7
Partner came to the facility within days 3–7	19	7.7
Partner came to the facility within days 8–14	27	10.9
Partner came to the facility 15+ days	49	19.7
Total	248	100.0
Average number of contacts to partners (missing information = 1)	Mean [range]	
Successfully referred (n = 248)	2 [1–5]	
Not successfully referred (n = 190)	1 [1–4]	

## Discussion

This study examined the acceptability, feasibility, and effectiveness of a partner notification and referral approach to HTS, an approach that has proven to be highly effective in identifying persons with undiagnosed HIV infection [9–11], but which has been underutilized in SSA. Aiming to build on a growing evidence base from countries in the region, we enrolled newly diagnosed HIV+ men and women as index clients at three hospitals in Njombe region, Tanzania’s highest HIV prevalence region, in which 14.8% of the adult population is infected with HIV [21].

The current study demonstrated high acceptability, feasibility, and effectiveness of this approach in the “real world” setting of routine facility-based HTS in Tanzania. High acceptability was evidenced by high uptake of the passive notification and referral process service among eligible index clients. A high level of feasibility was demonstrated, with more than half of listed sexual partners (56.6%) coming in for testing. The approach also proved to be effective; nearly 62% of successfully referred sexual partners were found to be HIV+, and of these, all were newly diagnosed. More than 70% of the HIV+ partners were linked to care and treatment. Our study found partner notification to be particularly effective in bringing current sexual partners in stable relationships (marriage or cohabitation) to the facility for testing. No cases of notification-related violence were reported in this study.

Our findings underscore the need for a good counseling and prevention package to be provided for serodiscordant couples as part of a partner notification program. Over one-third (36.2%) of the partners tested, all of whom indicated that they were in current partnership with the index client, tested negative. In our study, these couples were offered the standard of care in Tanzania during this time period, which included prevention counseling and condoms. Program and policy implementers should consider offering an effective prevention package for serodiscordant couples identified through partner notification approaches. This may include immediate initiation of ART for HIV+ partners. Additionally, pre-exposure prophylaxis (PrEP) for negative partners, where negative partners can come off PrEP if their positive partner is virally suppressed and they don’t have any other HIV risk, could be an important part of a serodiscordant couple package.

This study reinforces an emerging evidence base that in Africa, partner notification yields high rates of successful referral and high HIV positivity. In Kenya, in the first cluster randomized trial of partner notification in SSA, index clients were provided with an immediate provider-assisted partner notification service and 76% of their sexual partners were successfully referred to HTS [20]. Studies in Malawi and Cameroon have shown high HIV positivity rates among partners (64 and 50%, respectively), and as in Tanzania, the listed partner was generally a spouse or the main sexual partner of the index client [10, 11]. In a pilot study in Mozambique [17], community health workers provided assisted partner notification via contract referral to people newly diagnosed with HIV, and 54% of sexual partners were HIV+.

By assessing feasibility as well as effectiveness, this study offers unique insights into the application of partner notification in facility settings. Because index clients were offered a choice of referral method, rather than being randomized into a referral approach, we were able to assess index client preferences. The findings—that index clients overwhelmingly preferred passive referral and predominantly chose to list and notify a spouse—have important implications for the application and rollout of partner notification. There is clearly room for success in application of both client and provider initiated approaches to partner notification. In Kenya, 67% of sexual partners contacted using via provider-assisted partner notification came in for testing, when offered the service early [20]. Passive referral had not been highlighted as a promising approach in other studies: only 6.7% of partners in the Cameroon study were notified by passive referral [11]; in the Malawi study, passive referral had a comparatively poor uptake of 24% compared to 51% in the provider-assisted arms [10]. Our study was not designed to assess uptake of or otherwise make comparisons between referral methods, but rather to evaluate the effectiveness of a partner notification intervention implemented by providing a choice of referral by the index client. This would reflect a real world application of increasing focus on voluntary partner services into PITC/VCT contexts. Our index clients showed a much higher preference for passive referral. Relative to the Malawi study, the higher success of passive referral may be due to the fact that index clients were allowed to choose—rather than being randomized into—a notification approach; however, in the Cameroon study, index clients were allowed to choose their referral method and only a small proportion chose passive referral. The choices made by participants in our study suggest that, in Njombe, the majority of index clients were comfortable with passive referral, and viewed the role of the counselor as someone who could assist with facilitated disclosure of their status to their primary, current partner once the index client had convinced the partner to come for testing. Given the different results seen in this study and other studies in the region, implementers of partner notification approaches in SSA may wish to conduct formative research to explore preferences around provider-assisted versus passive referral, to create the most effective service delivery option.

Roughly 60% of the newly diagnosed individuals approached for enrollment in this study met eligibility criteria and elected to enroll. The most common reason for exclusion (n = 167, 65%) was not having a sexual partner in the last 24 months. In reality this may have been a response which allowed people who were anxious about partner notification to opt out of the process without stating their reluctance; this should be further investigated in future studies. These findings may differ in settings where HIV prevalence is lower, which may result in more stigma for HIV+ individuals and more reluctance to disclose status to partners. One limitation of the study was that we did not actively follow index clients or partners for IPV reporting. We were only able to ask about IPV from 20 index clients and 20 sexual partners who were interviewed 2–4 weeks following the partner notification process.

## Conclusions

Reaching the first 90 requires efficient and effective HIV testing strategies. As the proportion of PLHIV who remain undiagnosed decreases, reaching those who are asymptomatic and not engaged with the health system is a critical challenge.

Our study confirms that partner notification could dramatically increase the number of previously undiagnosed PLHIV who learn their status and are linked to care. Offering partner notification from within existing facility HTS settings could—with limited additional burden on the health system—greatly expand access to testing and linkage to care among people at very high risk of infection. Allowing index clients to choose their preferred referral method may have led to increased success in the referral process, resulting in more partners being tested. We found a clear preference for passive referral, especially to notify a spouse, among index clients in our study.

We recommend partner notification as a priority HIV testing strategy, and that provision of a package for prevention for serodiscordant couples be included as part of the service. Because of the heterogeneity in the successes and preferences associated with partner notification in different studies, no single partner notification strategy stands out as the recommended approach. However, our findings suggest that offering index clients options for passive or provider-facilitated notification and referral may result in a high uptake of passive referral. Further research is needed to evaluate whether or not partner notification strategies, tailored differently, could be more successful in reaching multiple or casual partners.

## Abbreviations

ART: Antiretroviral therapy
HTS: HIV testing services
IPV: Intimate partner violence
PITC: Provider initiated testing and counseling
STI: Sexually transmitted infections
VCT: Voluntary counseling and testing
